# Cavity piezo-mechanics for superconducting-nanophotonic quantum interface

**DOI:** 10.1038/s41467-020-17053-3

**Published:** 2020-06-26

**Authors:** Xu Han, Wei Fu, Changchun Zhong, Chang-Ling Zou, Yuntao Xu, Ayed Al Sayem, Mingrui Xu, Sihao Wang, Risheng Cheng, Liang Jiang, Hong X. Tang

**Affiliations:** 1 Department of Electrical Engineering, Yale University, New Haven, CT 06520 USA; 2 Department of Applied Physics, Yale University, New Haven, CT 06520 USA; 3 Yale Quantum Institute, Yale University, New Haven, CT 06520 USA; 4 Present Address: Center for Nanoscale Materials, Argonne National Laboratory, Argonne, IL 60439 USA; 5 Present Address: Pritzker School of Molecular Engineering, University of Chicago, Chicago, IL 60637 USA

**Keywords:** Optomechanics, Photonic devices, Superconducting devices, Quantum physics

## Abstract

Hybrid quantum systems are essential for the realization of distributed quantum networks. In particular, piezo-mechanics operating at typical superconducting qubit frequencies features low thermal excitations, and offers an appealing platform to bridge superconducting quantum processors and optical telecommunication channels. However, integrating superconducting and optomechanical elements at cryogenic temperatures with sufficiently strong interactions remains a tremendous challenge. Here, we report an integrated superconducting cavity piezo-optomechanical platform where 10 GHz phonons are resonantly coupled with photons in a superconducting cavity and a nanophotonic cavity at the same time. Taking advantage of the large piezo-mechanical cooperativity (*C*_em_ ~7) and the enhanced optomechanical coupling boosted by a pulsed optical pump, we demonstrate coherent interactions at cryogenic temperatures via the observation of efficient microwave-optical photon conversion. This hybrid interface makes a substantial step towards quantum communication at large scale, as well as novel explorations in microwave-optical photon entanglement and quantum sensing mediated by gigahertz phonons.

## Abstract (web summary)

Hybrid quantum systems would allow interfacing superconducting nodes with optical links. Here, the authors demonstrate an integrated platform where 10 GHz phonons are resonantly coupled with photons in a superconducting cavity and a nanophotonic cavity at the same time, allowing efficient mediation of bidirectional microwave-optical conversion.

## Introduction

Combining the most advanced technologies in different regimes, hybrid quantum architectures are pivotal for the development of quantum information science^1,2^. Recent few years have witnessed impressive progresses in two very important fields—quantum computing based on the state-of-the-art superconducting qubit technology^3–8^ and quantum telecommunication through low-loss optical photons^9–12^. However, toward a future scalable quantum network, it is becoming increasingly urgent to interface the superconducting and the photonic modalities in a quantum coherent manner^13,14^. To fully exploit the quantum advantages, such coherent superconducting-photonic interface must satisfy very stringent requirements: the signal interconversion must have not only a high efficiency but also low added noise.

Various physical platforms have been investigated to address these challenges in all aspects, including trapped irons or atoms^15,16^, magnonics^17^, electro-optics^18–21^, optomechanics^22–33^, etc. Among all the schemes, the optomechanical system is very appealing because strong photon–phonon coupling can be achieved in both microwave and optical domains to mediate efficient photon conversion. Benchmark demonstration of high-efficiency conversion has been recently achieved using a megahertz cavity electro-optomechanical system^32,33^. But the low-frequency mechanical membrane inevitably suffers from large thermal noise even at millikelvin temperatures, and the 3D optical cavity design poses a limit to the scalability.

On the other hand, gigahertz piezo-optomechanics^30,34–40^ is advantageous in that thermal excitations (${\bar{n}}_{{\rm{th}}}$) at high frequencies are significantly suppressed, ${\bar{n}}_{{\rm{th}}}\approx \frac{{k}_{{\rm{B}}}T}{\hslash \omega }$, where *k*_B_ is the Boltzmann constant, *ℏ* is the reduced Planck constant, *ω* and *T* are the frequency and the temperature. Moreover, these high-performance piezo-optomechanical (POM) micro-devices can be fully fabricated on a chip, offering great potential for integration and up-scaling.

However, it remains a tremendous challenge to efficiently interface an on-chip POM system with superconducting circuits. During past few years, great efforts have been made toward this goal. Bidirectional coherent conversion has been observed in room-temperature experiments by using a gigahertz POM crystal^30,41^. Recently, such a POM crystal has been cooled to its ground state for low-noise microwave-to-optical conversion^42^. Nevertheless, due to the lack of low-dissipation cavity-enhanced microwave coupling, in all existing realizations of POM converters, the achieved photon conversion efficiency is very limited (~10^−5^ at room temperature^41^ and  ~10^−10^ at cryogenic temperature^42^). Although superconducting cavity electromechanics has accomplished great successes^43–45^, combining superconducting and photonic devices is extremely difficult since superconductor absorbs light and light breaks superconductivity. It becomes even more challenging to integrate a superconducting cavity in the vicinity of a suspended optomechanical resonator with large mode overlap and minimized mode volume for enhanced interaction. Only a few potential designs have been proposed theoretically^28,46^, but no experimental realization has yet been achieved.

In this work, we demonstrate a triply resonant superconducting cavity POM interface that enables efficient microwave-optical (M-O) photon conversion. To our knowledge, this is the first realization of a superconducting POM converter that permits simultaneous cavity enhancement of photon–phonon interactions in both microwave and optical domains. By integrating a frequency-tunable superconducting cavity with a 10-GHz POM micro-disk, we are able to substantially enhance the microwave photon–phonon interaction and achieve a large electromechanical cooperativity (*C*_em_ ~7)—about three orders of magnitude improvement compared to previous POM converter demonstrations^41^. Combined with the implementation of a pulsed optical pump scheme to simultaneously boost the optomechanical interaction, we demonstrate coherent photon and phonon interactions via the observation of efficient bidirectional M-O photon conversion. The demonstrated superconducting-nanophotonic interface would not only advance the development of scalable quantum information networks, but also incorporate the state-of-the-art superconducting quantum technologies in the optical domain for breakthroughs in hybrid quantum systems, such as entangled M-O photon pair generation^47^, quantum repeaters^48^, and quantum metrology and sensing^49^.

## Results

### Hybrid cavity piezo-mechanics

**Fig. 1:**
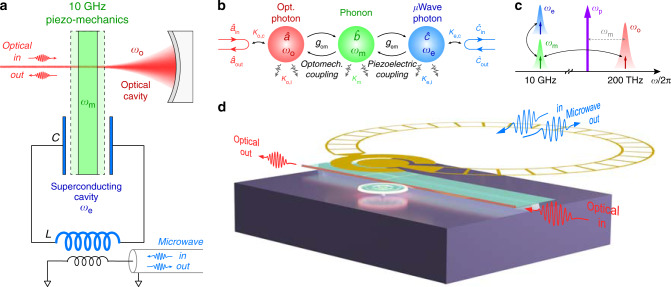
Superconducting cavity piezo-optomechanical system. **a** A schematic of a 10-GHz mechanical resonator simultaneously coupled with an optical cavity and a superconducting cavity to achieve resonantly enhanced optomechanical and electromechanical interactions at the same time. The superconducting cavity is inductively coupled to a transmission line for microwave signal input and output. **b** Interaction diagram of the triple-resonance system. *g*_em_ and *g*_om_ denote the electromechanical and the cavity-enhanced optomechanical coupling rates, respectively. **c** Photon conversion mechanism in the frequency domain. The blue, green, and red Lorentzian shapes stand for the microwave, mechanical, and optical resonances, respectively. A red-detuned optical pump is indicated as the purple arrow. **d** Experimental realization of the triply resonant superconducting piezo-optomechancial interface (schematic not to scale). A frequency-tunable superconducting “Ouroboros” microwave resonator (yellow) is aligned and coupled with a piezo-optomechanical micro-disk through the piezoelectric effect. Microwave and optical photons are interconverted via cavity-enhanced interactions with 10-GHz phonons supported by the thickness mode of the micro-disk.

The concept of our triply resonant superconducting POM system is illustrated in Fig. 1a. A high-frequency piezo-mechanical resonator at 10 GHz is coupled to an optical cavity through radiation pressure force. At the same time, the mechanical mode is exposed in the electric field from the capacitor of an superconducting LC resonator. As a result, cavity-enhanced piezoelectric interaction between the mechanical and the microwave modes can be achieved. The interaction diagram of the system is shown in Fig. 1b, where $\hat{a},\hat{b},\hat{c}$ are the annihilation operators of the optical, the mechanical, and the microwave modes, respectively, with resonant frequencies *ω*_o_, *ω*_m_, and *ω*_e_. The intrinsic dissipation rates of the three modes are denoted by *κ*_o,i_, *κ*_m_, and *κ*_e,i_. Input and output photons are coupled to the optical and the microwave modes with respective external coupling rates *κ*_o,c_ and *κ*_e,c_, contributing to the total dissipation rates *κ*_o_ ≡ *κ*_o,i_ + *κ*_o,c_ and *κ*_e_ ≡ *κ*_e,i_ + *κ*_e,c_ of the two modes.

The cavity piezoelectric interaction is characterized by a linear coupling rate *g*_em_, determined by the overlap between the microwave and the mechanical modes^44^. For M-O photon conversion, an optical pump tone is needed to compensate the photon energy difference. As illustrated in Fig. 1c, when the pump (purple arrow) is red-detuned from the optical resonance by ~*ω*_m_, the upper-sideband photon scattering (Stokes) is significantly enhanced by the optical mode so that efficient coupling between optical photons and phonons can be obtained with a linearized coupling rate given by ${g}_{{\rm{om}}}=\sqrt{{n}_{{\rm{cav}}}}{g}_{{\rm{om}},0}$. Here, *g*_om,0_ is the single-photon coupling rate and *n*_cav_ is the intra-cavity photon number populated by the pump. Combining with the cavity electromechanical coupling, efficient bidirectional conversion between microwave (~10 GHz) and optical photons (~200 THz) can be achieved.

In the resolved-sideband limit (*ω*_m_ ≫ *κ*_o_), the pump induced parametric amplification (anti-Stokes) can be neglected, and hence the system Hamiltonian can be expressed as (in the pump rotating frame)

1 $$\begin{array}{l}H/\hslash \ = -{\Delta }_{{\rm{o}}}{\hat{a}}^{\dagger }\hat{a}+{\omega }_{{\rm{m}}}{\hat{b}}^{\dagger }\hat{b}+{\omega }_{{\rm{e}}}{\hat{c}}^{\dagger }\hat{c} \hskip5.8pc\\ \ -{g}_{{\rm{om}}}({\hat{a}}^{\dagger }\hat{b}+{\hat{b}}^{\dagger }\hat{a}) -{g}_{{\rm{em}}}({\hat{b}}^{\dagger }\hat{c}+{\hat{c}}^{\dagger }\hat{b}),\end{array}$$

where Δ_o_ ≡ *ω*_p_ − *ω*_o_ is detuning of the pump frequency *ω*_p_ from the optical resonance. When the pump is detuned by Δ_o_ = − *ω*_m_, the maximum photon number conversion efficiency can be obtained (at *ω* = *ω*_m_; see Supplementary Note 5)

2 $${\eta }_{0}=\frac{{\kappa }_{{\rm{e}},{\rm{c}}}}{{\kappa }_{{\rm{e}}}}\frac{{\kappa }_{{\rm{o}},{\rm{c}}}}{{\kappa }_{{\rm{o}}}}\frac{4{C}_{{\rm{em}}}{C}_{{\rm{om}}}}{{({C}_{{\rm{em}}}+{C}_{{\rm{om}}}+1)}^{2}+4\frac{{({C}_{{\rm{om}}}+1)}^{2}}{{\kappa }_{{\rm{e}}}^{2}}{\delta }_{{\rm{em}}}^{2}}.$$

Here, ${C}_{{\rm{em}}}\equiv \frac{4{g}_{{\rm{em}}}^{2}}{{\kappa }_{{\rm{e}}}{\kappa }_{{\rm{m}}}}$ and ${C}_{{\rm{om}}}\equiv \frac{4{g}_{{\rm{om}}}^{2}}{{\kappa }_{{\rm{o}}}{\kappa }_{{\rm{m}}}}$ are the electromechanical and the optomechanical cooperativities, respectively. *δ*_em_ ≡ *ω*_e_ − *ω*_m_ is the frequency difference between the microwave and the mechanical resonances. To achieve the highest efficiency, ideally, the microwave and the mechanical modes should be on resonance (*δ*_em_ = 0), and large and matched cooperativities (*C*_em_ = *C*_om_ ≫ 1) with very over-coupled readout ports ($\frac{{\kappa }_{{\rm{e}},{\rm{c}}}}{{\kappa }_{{\rm{e}}}},\frac{{\kappa }_{{\rm{o}},{\rm{c}}}}{{\kappa }_{{\rm{o}}}}\to 1$) are desired. In a POM system, large optomechanical cooperativity has been previously demonstrated^34,42^. However, so far the achieved electromechanical cooperativity is only ~4 × 10^−3^ (Jiang et al.^41^) due to the lack of microwave cavity enhancement, which has become the main efficiency bottleneck of POM converters.

### Triple-resonance integration

We address this challenge and experimentally realize the triply resonant system by integrating a POM micro-disk and a planar superconducting microwave resonator as depicted in Fig. 1d. The micro-disk simultaneously supports a high-quality (*Q*) factor optical whispering gallery mode and a high-frequency mechanical thickness mode at 10 GHz^36^. To maximize the electromechanical mode overlap and coupling, we implement our frequency-tunable “Ouroboros” design of the superconducting resonator^50^. The “Ouroboros” (yellow) forms a planar LC resonator which concentrates the electric field around its capacitor pads. Aligning the micro-disk in the close vicinity under the capacitor pad of the “Ouroboros” allows maximized overlap between the perpendicular electric field of the microwave mode and the strain field of the mechanical thickness mode. As a result, cavity-enhanced electromechanical coupling is achieved. For converter operation, optical photons are sent into and read out from the micro-disk through an on-chip coupling waveguide; microwave input/output photons are inductively coupled to the “Ouroboros” by using a loop probe.

**Fig. 2:**
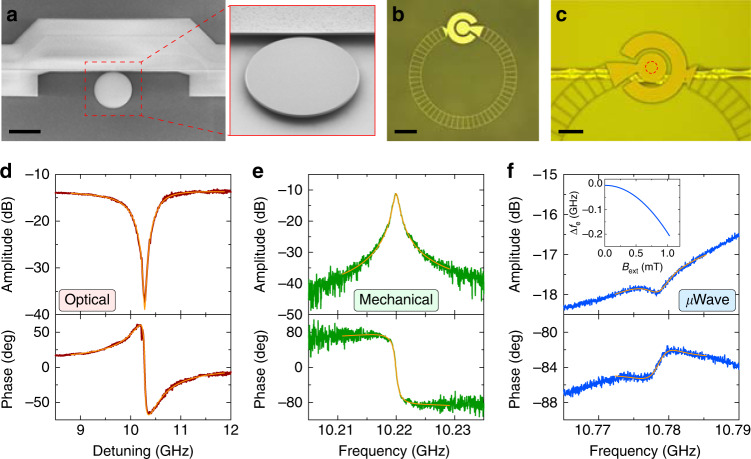
Device design and characterization. **a** An SEM image of the AlN piezo-optomechanical micro-disk resonator with a radius of 12 μm. An angled zoomed-in view of the suspended disk is shown on the right. **b** An optical image of the frequency-tunable superconducting “Ouroboros” microwave resonator fabricated in NbN on sapphire substrate. **c** An optical image of the integrated device showing the alignment between the “Ouroboros” and the micro-disk. The “Ouroboros” is flipped over with its circular capacitor pad aligned above the micro-disk (indicated in a red dashed circle). Scale bars in **a**–**c** are 20, 100, and 50 μm, respectively. **d**–**f** Spectrum of the optical, the mechanical, and the microwave resonances, respectively, with both amplitude and phase response at 900 mK. Orange lines are Lorentzian fittings. The detuning in **d** is from a pump laser at 194.363 THz (1543.5 nm). The inset in **f** shows the resonant frequency tuning of the “Ouroboros” under an external magnetic field. The resonance in **f** is at zero field.

An SEM image of the micro-disk is shown in Fig. 2a, fabricated in aluminum nitride (AlN) with a thickness of 550 nm and a radius of 12 μm. The micro-disk is suspended and supported by a silicon dioxide anchor on top of a high-resistivity silicon substrate. The size of the anchor tip is minimized (<100 nm in radius) by precise fabrication control for reducing acoustic radiation loss. Figure 2b shows an optical image of the superconducting “Ouroboros” resonator fabricated in a 50-nm-thick niobium nitride (NbN) film on a thin sapphire substrate. In order to better align resonant frequency of the “Ouroboros” with the thickness mode of the micro-disk, hole array structure is fabricated in the inductor wire of the “Ouroboros” to realize frequency tunability by modifying its kinetic inductance via an external magnetic field^50^. After separate chip fabrication, the “Ouroboros” is flipped over and aligned above the micro-disk with ~5-μm spacing (Fig. 2c). The two chips are then bonded together, and two optical fibers are aligned and attached to the side-coupled optical waveguide of the micro-disk using epoxy. Device fabrication details are described in Methods and Supplementary Note 1.

The integrated superconducting POM device is loaded in a dilution refrigerator on the still flange (900 mK) for measurement. Although the ~1-K environment presents slightly higher thermal noise (${\bar{n}}_{{\rm{th}}}\approx 1.6$), it provides much larger cooling power and thermal conductivity compared with millikelvin environment, which allows higher optical pump power for efficient photon conversion. Moreover, it is possible to radiatively cool a gigahertz mode and suppress its thermal occupancy for quantum operations^47^ in spite of a hotter physical temperature of the resonator. We have recently demonstrated such radiative cooling of an “Ouroboros” mode by constructing a cooling channel to a millikelvin cold bath^51,52^. In this work, we focus on the demonstration of coherent M-O photon conversion in our triply resonant POM system; systematic characterization of the noise performance as well as the implementation of radiative cooling is subject to future investigation.

The spectra of the optical, the mechanical, and the microwave resonances of the hybrid device are shown in Fig. 2d–f, respectively, measured at 900 mK. The optical resonance is characterized using a single-sideband modulation scheme to reveal the phase response (details are explained later). The fiber-to-fiber optical transmission efficiency is measured to be 4.3%, where the 14-dB loss mainly comes from the imperfect mode matching at the fiber-to-chip interfaces. The optical intrinsic and coupling *Q* factors are fitted to be *Q*_o,i_ = 5.4 × 10^5^ and *Q*_o,c_ = 5.8 × 10^5^, respectively, corresponding to $\frac{{\kappa }_{{\rm{o}},{\rm{i}}}}{2\pi }=0.36\ {\rm{GHz}}$ and $\frac{{\kappa }_{{\rm{o}},{\rm{c}}}}{2\pi }=0.34\ {\rm{GHz}}$. The mechanical resonance of the micro-disk is characterized by electro-optomechanical driven response—similar to our previous room-temperature measurement scheme described in Han et al.^36^. The thickness mode of the micro-disk is observed at $\frac{{\omega }_{{\rm{m}}}}{2\pi }=10.220\ {\rm{GHz}}$ with *Q*_m_ = 1.1 × 10^4^ ($\frac{{\kappa }_{{\rm{m}}}}{2\pi }=0.93\ {\rm{MHz}}$). The “Ouroboros” resonance at zero external magnetic field is measured to be $\frac{{\omega }_{{\rm{e}}}}{2\pi }=10.778\ {\rm{GHz}}$ with *Q*_e,i_ = 2.6 × 10^3^ ($\frac{{\kappa }_{{\rm{e}},{\rm{i}}}}{2\pi }=4.1\ {\rm{MHz}}$) and *Q*_e,c_ = 9.9 × 10^4^ ($\frac{{\kappa }_{{\rm{e}},{\rm{c}}}}{2\pi }=0.11\ {\rm{MHz}}$). This under-coupling of the microwave port is caused by the nonideal faraway position of the loop probe from the “Ouroboros”. By applying an external magnetic field, more than 200 MHz frequency tuning range of the “Ouroboros” resonance is realized without obvious degradation of the quality factor (inset in Fig. 2f). The single-photon optomechanical coupling rate is fitted to be $\frac{{g}_{{\rm{om}},0}}{2\pi }\approx 19\ {\rm{kHz}}$ (details are explained in the last section). Benefited from the triple-resonance design, a high electromechanical coupling rate $\frac{{g}_{{\rm{em}}}}{2\pi }\approx 2.7\ {\rm{MHz}}$ is achieved (see Supplementary Note 2), resulting in a large cooperativity *C*_em_ ~7.

### Pulsed photon conversion

**Fig. 3:**
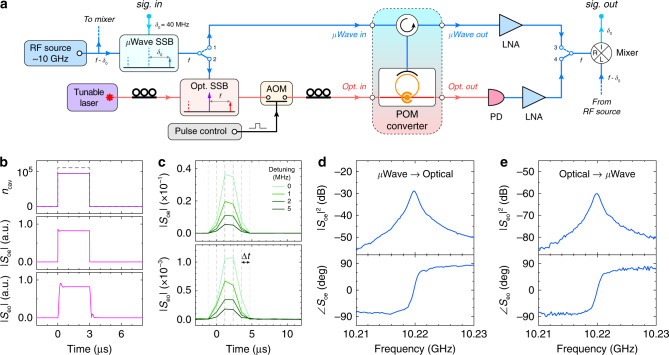
Pulsed microwave-optical photon conversion. **a** A schematic of measurement scheme with pulsed optical pump. A 3-μs-wide red-detuned pump pulse is generated by on/off switching using an acoustic-optic modulator (AOM). To characterize the device temporal response, a low-frequency microwave signal (*δ*_0_ = 40 MHz) from a lock-in amplifier (not shown here) is upconverted to *f* ~ 10 GHz around the mechanical resonance by single-sideband (SSB) modulation. The signal is then sent to the device either as the microwave input (switch pos. 1), or the optical input after the optical SSB generation (pos. 2). The microwave or optical output (pos. 3 and 4, respectively) is then downconverted to 40 MHz and sent to the lock-in amplifier to measure the quadratures in time domain. PD: photodetector, LNA: low-noise RF amplifier. **b** Theoretical calculation of the temporal response of the system under a pulsed pump (profile indicated in gray dashed line in the top panel). *n*_cav_: intra-cavity photon number. *S*_oe_ and *S*_eo_: microwave-to-optical and optical-to-microwave conversion signal, respectively. **c** Experimental data of the pulsed conversion signals at different input detunings from the mechanical resonance ($\frac{{\omega }_{{\rm{m}}}}{2\pi }=10.220\ {\rm{GHz}}$). The measurement time resolution is Δ*t* ≈ 1.17 μs. **d**, **e** Coherent bidirectional microwave-optical photon conversion spectra with both amplitude and phase responses.

To realize efficient M-O photon conversion, a large optomechanical cooperativity is equally important. We exploit a pulsed-pump scheme to reduce the heating effect^42,53^ and boost the intra-cavity photon number *n*_cav_. The measurement configuration is illustrated in Fig. 3a. A continuous-wave (c.w.) pump tone (purple arrow) is provided by a stable tunable laser, red-detuned by Δ_o_ = −*ω*_m_ from the optical resonance at 1543.420 nm. The pump light is then pulsed by on/off switching using an acoustic-optic modulator (AOM). In order to characterize the coherent response of the POM system in time domain, a low-frequency (*δ*_0_ = 40 MHz) signal (labeled as sig. in in Fig. 3a) from a lock-in amplifier is upconverted to  ~10 GHz via microwave single-sideband (SSB) modulation technique. The upconverted c.w. microwave probe tone (blue arrow) can either be directly sent to the converter (switch pos. 1) as its microwave input, or generate the optical input (red arrow) through an optical SSB modulator (switch pos. 2). The microwave or the optical output (switch pos. 3 or 4) is then downconverted to 40 MHz and sent back to the lock-in amplifier for amplitude and phase measurement in time domain. Detailed experimental setup is explained in Methods and Supplementary Note 3.

Due to the finite temporal resolution of the lock-in amplifier (Δ*t* ≈ 1.17 μs), we set a minimum pump pulse width of 3 μs with a repetition period of 1 ms. Theoretical analysis indicates that, due to the fast dynamical response, the conversion process can be treated as a quasi-steady state during the pulse operation. Numerical calculations of the system response are plotted in Fig. 3b. The top panel shows the rapid population of intra-cavity optical photons under an ideal 3-μs pump; the fast response time corresponds to the optical cavity decay rate 1/*κ*_o_ ≈ 0.2 ns. In experiment, the rise-/fall-time of the pump pulse and *n*_cav_ is determined by the AOM switching speed (<35 ns), which is still much shorter than the pump pulse width. The middle and the bottom panels show the microwave-to-optical (*S*_oe_) and the optical-to-microwave (*S*_eo_) conversion signal, respectively. In both cases, the transient response time is less than a few hundreds of nanoseconds, which is determined by the coupling and the dissipation rates *g*_om_, *g*_em_, *κ*_e_, *κ*_m_ ~ MHz × 2*π* (see Supplementary Note 5 for analysis). Figure 3c shows typical temporal M-O conversion pulses measured in experiment at different input detunings from the mechanical resonance. It can be seen that, when the pump is turned on, conversion signals are clearly observed in both directions with the maximum magnitude at zero detuning. By sweeping the input frequency, bidirectional M-O photon conversion spectra are obtained in Fig. 3d and e with both amplitude and phase responses, confirming the coherence of the conversion process. A large conversion bandwidth of 1 MHz is achieved around the center mechanical resonant frequency.

### Conversion efficiency and thermal shift

**Fig. 4:**
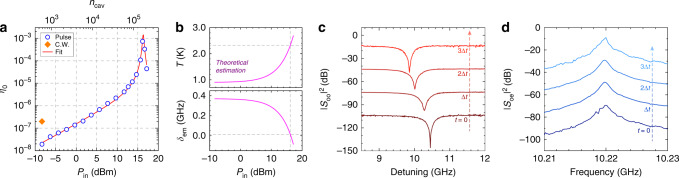
Conversion efficiency and thermal shift. **a** Calibrated on-chip microwave-optical photon conversion efficiency *η*_0_ at different optical pump power *P*_in_. **b** Estimated temperature *T* and electromechanical resonance mismatch *δ*_em_ of the “Ouroboros” at different pump powers. Gray dashed lines label the values at *P*_in_ = 16 dBm when the highest conversion efficiency is measured. **c**, **d** The optical resonance and the conversion spectrum at different time during a 16-dBm pump pulse, respectively. The spectra are vertically displaced for comparison purpose. The time resolution is Δ*t* ≈ 1.17 μs.

The photon conversion efficiency can be calibrated by measuring the full spectra of the scattering matrix elements. Here, we use *S*_*ij*_[*ω*] (*i*, *j* = o, e) to denote the directly measured reflection or conversion spectra, which are proportional to intrinsic scattering matrix elements *t*_*ij*_[*ω*] of the converter (within the dashed box in Fig. 3a) up to a constant gain or loss factor. Since the system is reciprocal, the gain and loss factors along each input and output path can be calibrated out together to obtain the conversion efficiency $\eta [\omega ]={\left|{t}_{{\rm{oe}}}[\omega ]\right|}^{2}={\left|{t}_{{\rm{eo}}}[\omega ]\right|}^{2}$ (see Supplementary Note 4), which simplifies to Eq. (2) at the peak of the spectrum (*η*[*ω*_m_] = *η*_0_). The on-chip M-O photon conversion efficiencies *η*_0_ at different optical pump powers *P*_in_ are plotted as blue circles in Fig. 4a. The corresponding intra-cavity photon number *n*_cav_, which is proportional to *P*_in_, is labeled on the upper *x*-axis. It can be seen that at low powers, *η*_0_ increases linearly with *P*_in_ and *n*_cav_, as expected from Eq. (2) when *C*_om_ ≪ 1. As the pump power further increases, the efficiency dramatically goes up to a peak value and then drops. This behavior can be attributed to the pump induced thermal shift of the microwave resonance as discussed below. The highest efficiency achieved is (7.3 ± 0.2) × 10^−4^, which corresponds to an internal efficiency of (5.8 ± 0.2)% after excluding the low extraction factor ($\frac{{\kappa }_{{\rm{o}},{\rm{c}}}}{{\kappa }_{{\rm{o}}}}\frac{{\kappa }_{{\rm{e}},{\rm{c}}}}{{\kappa }_{{\rm{e}}}}\approx 1.3 \%$).

The thermal effect can be observed from the optical resonance shift during the pump pulse. Figure 4c shows the optical spectrum at different time within the pulse at a pump power of 16 dBm. It can be seen that after the pump is turned on, the optical resonance gradually shifts to lower frequencies by ~0.59 GHz within the 3-μs pulse. Therefore, in our experiment, the detuning of the optical pump is always adjusted and optimized especially at high powers to compensate the thermal shift and achieve the highest conversion efficiency. In contrast, the thermal effect has negligible influence on the mechanical resonance since the small relative frequency shift (<10^−5^) corresponds to <0.1 MHz at the mechanical frequency, which is much smaller than the mechanical linewidth and hence not noticeable in the conversion spectrum (Fig. 4d).

In our POM system, the “Ouroboros” has an original resonance at 10.778 GHz, higher than the mechanical resonance of the micro-disk at 10.220 GHz. Therefore, an external magnetic field (~1 mT) is applied to tuned the “Ouroboros” frequency down to 10.590 GHz to reduce the frequency mismatch. At low optical pump powers, the remained mismatch (*δ*_em_ in Eq. (2)) compromises the electromechanical interaction and hence limits the conversion efficiency. Nevertheless, at high pump powers, the heating effect (mostly comes from the fiber-to-chip interface in our case) becomes non-negligible, and the “Ouroboros” frequency will further decrease due to the reduction of Cooper pair density in the superconductor. As a result, raising the pump power will not only boost the optomechanical cooperativity *C*_om_, but also reduce the electromechanical resonance mismatch *δ*_em_, thus giving rise to a sharp increase of the conversion efficiency.

Due to the under-coupling condition of the microwave probe and the imperfect measurement background, it is difficult to trace the “Ouroboros” resonance at high pump powers. However, we can estimate the frequency shift and the temperature of the “Ouroboros” based on the pump power dependence of the conversion efficiency. Because the inductance of the “Ouroboros” is dominated by the kinetic inductance (*L*_k_), we assume that its resonance frequency shift is affected by temperature primarily via ${\omega }_{{\rm{e}}}(T)\propto \frac{1}{\sqrt{{L}_{{\rm{k}}}(T)}}$. Using the temperature dependence of kinetic inductance from BCS theory^54^, we have

3 $${\omega }_{{\rm{e}}}(T)\approx {\nu }_{0}{\left[\sqrt{1-\frac{T}{{T}_{{\rm{c}}}}}\tanh \left(1.53\frac{{T}_{{\rm{c}}}}{T}\sqrt{1-\frac{T}{{T}_{{\rm{c}}}}}\right)\right]}^{1/2},$$

where *T*_c_ ≈ 12 K is the critical temperature of the NbN film, and *T* ≈ *T*_0_ + *βP*_in_ assuming that the temperature change from *T*_0_ = 900 mK is proportional to the pump power *P*_in_. *ν*_0_ and *β* are two proportional constants. *ν*_0_ can be determined by the initial Ouroboros frequency $\frac{{\omega }_{{\rm{e}}}(T)}{2\pi }=10.590$ GHz at *T* = *T*_0_, while *β* is treated as a fitting parameter.

Substituting Eq. (3) into Eq. (2), we can get the power dependence of conversion efficiency. By setting *β*, *g*_om,0_, and *g*_em_ as free parameters while using experimentally obtained values for other device parameters, the conversion efficiency can be fitted very well as indicated in the red line in Fig. 4a. The thermal constant and the single-photon optomechanical coupling rate are extracted to be *β* = (3.43 ± 0.02) × 10^−2^ K· mW^−1^ and $\frac{{g}_{{\rm{om}},0}}{2\pi }=(19\pm 2)$ kHz, respectively. The electromechanical coupling rate is fitted to be $\frac{{g}_{{\rm{em}}}}{2\pi }=(3.2\pm 0.3)$ MHz, which agrees well with our experimental characterization (~2.7 MHz, see Supplementary Note 2). The corresponding temperature and resonant frequency shift of the “Ouroboros” at different pump powers are plotted in Fig. 4b. Note that they represent the estimated values at the time point (*t* = 3Δ*t*) when the conversion efficiency in Fig. 4a is obtained. As expected, the temperature gradually goes up, whereas the resonant frequency decreases, with increasing pump power. At the highest measured conversion efficiency, the fitting indeed infers a close-to-zero electromechanical resonance mismatch ($\frac{{\delta }_{{\rm{em}}}}{2\pi }\approx 14$ MHz, gray dashed line in the lower panel of Fig. 4b). At zero mismatch, the fitting predicts a slightly higher peak efficiency of 1.4 × 10^−3^ (internal 11%), which is not resolved in experiment due to the finite resolution of the pump power sweep. The theoretical estimation also indicates that the “Ouroboros” temperature remains below ~3 K even at the highest pump power that we applied. This is reasonable since the thermally induced relative frequency shift of the superconducting “Ouroboros” is only ~4%. It is worth pointing out that the measured efficiency-pump power dependence in Fig. 4a is highly repeatable without observation of hysteresis effect (see Supplementary Note 4 for details). This confirms that during our measurement the “Ouroboros” always remains in its superconducting state with its temperature well below *T*_c_, even in presence of the external magnetic bias and the pump induced heating.

Therefore, by implementing the pulsed pump scheme, we are able to significantly reduce the optical heating and boost the pump photon number. The optomechanical cooperativity at the highest conversion efficiency is estimated to be *C*_om_ ~ 0.4. For comparison, we also perform measurement using c.w. optical pump. Because of the excessive heating during c.w. operation, the pump power that can be applied in the cryogenic environment is significantly constrained. Within the cooling capacity of our dilution refrigerator, the maximum c.w. pump power is limited to −8.3 dBm, which is over two orders of magnitude less than that can be applied in the pulsed scheme. As a result, the best c.w. conversion efficiency obtained is only 2 × 10^−7^ (orange diamond point in Fig. 4a).

## Discussion

Taking advantage of the integration of the superconducting microwave cavity, we demonstrated a large *C*_em_ ~7 in our triply resonant POM interface. To further improve the conversion efficiency, *C*_om_ needs to be enhanced to match *C*_em_. This could be done by either improving the optical and the mechanical quality factors through better fabrication process or further increasing the pump photon number. The pump induced heating would be reduced if the fiber-to-chip coupling loss is minimized.

Toward quantum-enabled M-O photon conversion, it is equally important to minimize the added noise induced by thermal excitations. The high operation frequency of our system is advantageous to suppress thermal noise from both microwave and mechanical baths. The estimated temperature of the “Ouroboros” leads to a thermal occupancy of the microwave bath around only a few photons (${\bar{n}}_{{\rm{the}}}\approx 5.6$ at 3 K). In Supplementary Note 6, we provide comprehensive theoretical analysis of the added noise during the photon conversion process in both directions. We show that it is possible to suppress both the microwave and the mechanical added noise to subphoton level even the device is physically in a relatively “hot” environment. This can be realized by implementing the radiative cooling scheme^51,52^ with an over-coupled microwave port. In addition, a large *C*_om_ is needed to suppress the mechanical added noise during the optical-to-microwave photon conversion. Future experimental characterization of the noise performance of photon conversion in our system will be valuable.

In summary, we have realized an integrated interface between superconducting and nanophotonic circuits. The demonstrated high-frequency superconducting cavity POM system provides an important route toward the development of scalable efficient M-O photon conversion devices. Combining with our recently demonstrated radiative cooling technique and feasible future improvement of device parameters, quantum operations braiding superconducting microwave, nanomechanics, and nanophotonics can be expected.

## Methods

### Device fabrication

Detailed device fabrication procedure is described in Supplementary Note 1. In brief, the POM micro-disk and the superconducting “Ouroboros” are first fabricated separately, then aligned and integrated by flip-chip bonding technique. The device patterns are defined using electron-beam lithography followed by dry etching processes. The micro-disk is released in buffered oxide etch to form the suspended structure. After the integration of the two chips, optical fibers are side-coupled and aligned to the on-chip coupling waveguide of the micro-disk, then secured using ultraviolet epoxy. The integrated device is finally enclosed in a copper box holder for low-temperature measurement.

### Measurement schemes

Detailed measurement setup is described in Supplementary Note 3, and the measurement repeatability and the efficiency calibration procedure are described in Supplementary Note 4. The POM micro-disk and the superconducting “Ouroboros” are characterized separately before they are integrated. The micro-disk is first measured at room temperature, while the “Ouroboros” is measured in liquid helium. After integration, the device is loaded in the dilution refrigerator for cryogenic experiments. Low-power c.w. measurements are first performed to obtain the optical, the mechanical, and the microwave resonance spectra. Then a pulsed optical pump is applied to investigate the M-O photon conversion as explained in the main text and Supplementary Note 3.

## Supplementary information

Supplementary Information

## Data Availability

The data that support the plots within this paper and other findings of this study are available from the corresponding author (H.X.T.) upon reasonable request.
