# Supplementary material for: Cavity piezo-mechanics for superconducting-nanophotonic quantum interface
Source: Nat Commun. 2020 Jun 26;11:3237. doi: 10.1038/s41467-020-17053-3 (PMC7320138; doi:10.1038/s41467-020-17053-3)
Supplement: Supplementary file 1 — Supplementary Information [file 41467_2020_17053_MOESM1_ESM.pdf]

Supplementary Information for "Cavity piezo-mechanics for superconducting-nanophotonic quantum interface"

Xu Han,[1,*] Wei Fu,[1] Changchun Zhong,[2,3,†] Chang-Ling Zou,[1] Yuntao Xu,[1] Ayed Al Sayem,[1] Mingrui Xu,[1] Sihao Wang,[1] Risheng Cheng,[1] Liang Jiang,[2,3,†] and Hong X. Tang[1,3,‡]

[1] *Department of Electrical Engineering, Yale University, New Haven, Connecticut 06520, USA*
[2] *Department of Applied Physics, Yale University, New Haven, Connecticut 06520, USA*
[3] *Yale Quantum Institute, Yale University, New Haven, Connecticut 06520, USA*

[*] Present address: Center for Nanoscale Materials, Argonne National Laboratory, Argonne, Illinois 60439, USA
[†] Present address: Pritzker School of Molecular Engineering, University of Chicago, Chicago, Illinois 60637, USA
[‡] hong.tang@yale.edu

Supplementary Note 1. Device fabrication

The POM micro-disk is fabricated in a 550-nm-thick c-axis aligned AlN layer on an oxidized high-resistivity silicon substrate. A 200-nm-thick silicon dioxide (SiO_2) layer is first deposited on top of the AlN as hard mask via plasma-enhanced chemical vapor deposition (PECVD). The photonic pattern is then defined using electron-beam lithography (EBL) with Ma-N 2403 resist, followed by resist reflow technique to improve surface smoothness. Fluorine-based reactive-ion etching (RIE) is performed to transfer the pattern in the SiO_2 mask layer. The AlN is then shallow-etched using chlorine-based RIE with a thin layer (~ 100 nm) left as the protection layer during the later releasing process. The second EBL is performed with ZEP 520A resist to define the releasing areas where the rest of the AlN is etched through by the second chlorine-based RIE. In order to integrate with the "Ouroboros" chip, PECVD SiO_2 and silicon nitride (SiN) layers (5 µm and 500 nm thick, respectively) are deposited successively around edge areas of the AlN chip as spacers besides the micro-disk. The SiN protects the SiO_2 spacers from being etched during the releasing process. The device is then released in buffered oxide etch (BOE) to etch the sacrificial SiO_2 layer beneath the AlN and suspend the micro-disk. The releasing process is precisely timed so that the SiO_2 anchor of the micro-disk has a minimized tip size around 100 nm in radius. The chip is finally cleaved to prepare for fiber side coupling at the two ends of the photonic waveguide.

The superconducting "Ouroboros" is fabricated in a 50-nm-thick NbN film deposited on a 150-µm-thick sapphire substrate. The "Ouroboros" structure is defined by EBL using hydrogen silsesquioxane (HSQ) resist. After individual fabrication and characterization of the micro-disk and the "Ouroboros", the two chips are aligned under a microscope and integrated together. The "Ouroboros" chip is flipped over and placed on top of the AlN chip with the circular capacitor pad of the "Ouroboros" aligned above the AlN micro-disk. The SiO_2 spacers maintain a $\sim$5-µm gap between the "Ouroboros" and the micro-disk so that optics and superconductivity would not compromise each other. This gap, on the other hand, is still much smaller than the microwave wavelength and mode size, which ensures large piezo-electromechanical coupling. The aligned chips are then bonded together by applying ultraviolet (UV) epoxy at the edges. Two ultra-high numerical aperture (UHNA) optical fibers with cleaved flat end surface are aligned to the two ends of the coupling waveguide of the micro-disk. The alignment is performed by precisely adjusting the positions of the fibers using motorized stages to maximize the optical transmission. The fibers are then secured to the chip using UV epoxy. The integrated superconducting POM device is then enclosed in a copper box holder for measurement. Only three close-fit ports of the box are left open for the microwave probe and two optical fibers. In this way, optimal shielding from the environmental noises and perturbations can be obtained. A home-made superconducting coil is placed outside the box holder above the "Ouroboros" to provide magnetic field for frequency tuning. The coil is made by winding a long niobium-titanium (NbTi) superconducting wire with its two terminals connected to the DC line breakout under the 4K plate of the dilution refrigerator. A DC current (less than a few tens of milli-Ampere) is sent to the coil to generate a magnetic field of 16 mT/A at the "Ouroboros" position.

Supplementary Note 2. Electromechanical coupling rate g_{em}

The electromechanical coupling rate g_{em} can be characterized by measuring the microwave reflection spectrum of the coupled modes. Since in our system, the mechanical resonance of the micro-disk is out of the frequency tuning range of the "Ouroboros", we experimentally characterize g_{em} using a separate device, fabricated in the same way but the "Ouroboros" has slightly lower resonant frequency which can be aligned with mechanical thickness mode of the micro-disk.

As shown in Fig. 1a, when the "Ouroboros" resonance is tuned to lower frequencies by the external magnetic field, avoided crossing is observed, indicating strong electromechanical coupling. Figure 1b plots the amplitude and phase spectrum at $B_{\mathrm{ext}} = 0.09$ mT (gray dashed line in (a)), which can be fitted by using the microwave reflection coefficient without the optomechanical coupling

$$S_{\mathrm{ee}}[\omega]\Big|_{g_{\mathrm{om}}=0} = -1 + \frac{\kappa_{\mathrm{e,c}}}{-i(\omega-\omega_{\mathrm{e}}) + \frac{\kappa_{\mathrm{e}}}{2} + \frac{g_{\mathrm{em}}^2}{-i(\omega-\omega_{\mathrm{m}})+\frac{\kappa_{\mathrm{m}}}{2}}}. \tag{S1}$$

From the fitting (red lines in Fig. 1b), we extract $\frac{g_{\mathrm{em}}}{2\pi} = 2.7$ MHz. The intrinsic microwave and mechanical Q factors and dissipation rates of this devices are $Q_{\mathrm{e,i}} = 1.7\times10^3$ ($\frac{\kappa_{\mathrm{e,i}}}{2\pi} = 6.4$ MHz) and $Q_{\mathrm{m}} = 1.4\times10^4$ ($\frac{\kappa_{\mathrm{m}}}{2\pi} = 0.74$ MHz).

It is worth pointing out that the slightly different resonant frequencies and Q factors of the device have negligible influence on the microwave and the mechanical mode profiles and their overlap; hence won't affect the electromechanical coupling. As discussed in the main text, this directly measured g_{em} from the characterization device agrees very well with the fitted value based on the power dependence of the photon conversion efficiency in our superconducting POM system. For a conservative estimation, we use $\frac{g_{\mathrm{em}}}{2\pi} = 2.7$ MHz and calculate $C_{\mathrm{em}} \approx 7.4$.

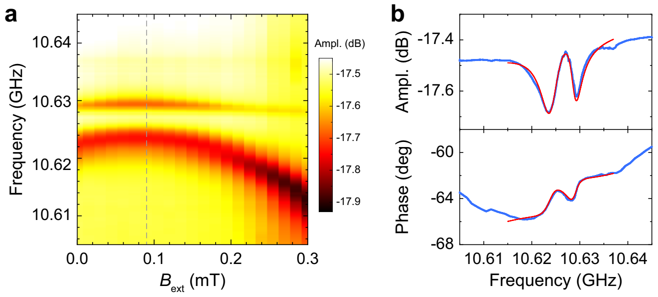

Supplementary Figure 1. **Measurement of the electromechanical coupling rate g_{em}. a** Microwave reflection spectrum at different external magnetic fields. As the "Ouroboros" resonance is tuned across the mechanical resonance of the micro-disk, avoided crossing is observed. **b** Line plot of the spectrum at $B_{\mathrm{ext}} = 0.09\,\mathrm{mT}$ (gray dashed line in **a**) with both amplitude and phase responses. The red lines are the fitting using coupled mode formula, which extracts a coupling rate $\frac{g_{\mathrm{em}}}{2\pi} = 2.7\,\mathrm{MHz}$.

Supplementary Note 3. Measurement setup

Detailed measurement setup of the pulsed microwave-optical photon conversion is shown in Fig. 2. An ultra-high-frequency lock-in amplifier (Zurich Instruments UHFLI) is used to send two low-frequency ($\delta_0 = 40\,\mathrm{MHz}$) signals to an IQ mixer (Marki IQ-0618LXP), with their phase difference fine-tuned around 90° for synthesizing microwave single sideband from an RF source (Anritsu MG3693C) at $\sim 10\,\mathrm{GHz}$ as local oscillator (LO). This upconverted single-sideband microwave tone can be swept by varying the frequency of the LO, and sent to either the converter (switch pos. 1) as a c.w. microwave input or an optical single-sideband modulator (SSBM, Fujitsu FTM7961) to generate an optical input (switch pos. 2). The optical single sideband is generated from a stable tunable laser (Santec TSL-710), which at the same time serves as the pump after amplified by an erbium-doped fiber amplifier (EDFA) and filtered by a tunable filter to reduce noise. This c.w. pump is pulsed by switching on/off an acoustic-optic modulator (AOM, Gooch & Housego T-M080). This is done by sending a 3-μs trigger pulse with a repetition period of 1 ms from a function generator to a TTL switch to modulate the 80-MHz RF carrier of the AOM. At the same time, the trigger pulse is split and sent to the Zurich UHFLI as the external trigger for temporal measurement. The output signal of the converter at $\sim 10\,\mathrm{GHz}$ is downconverted to 40 MHz using the same RF source and sent to the Zurich for detection in time domain.

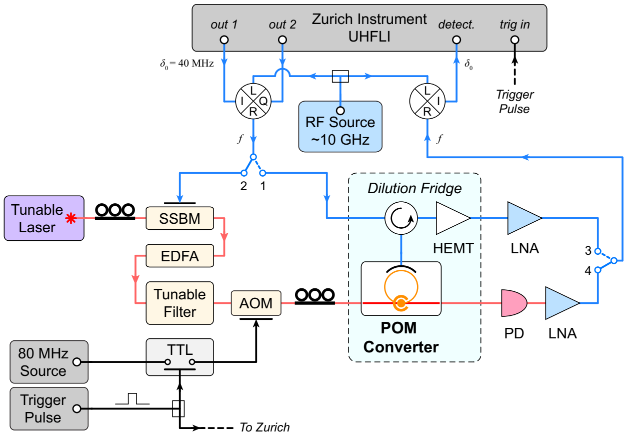

Supplementary Figure 2. **Measurement setup of the pulsed photon conversion.** UHFLI: ultra-high-frequency lock-in amplifier. SSBM: single-sideband modulator. EDFA: erbium-doped fiber amplifier. AOM: acoustic-optic modulator. TTL: transistor-transistor logic switch. HEMT: high-electron-mobility transistor amplifier. PD: photodetector. LNA: low-noise RF amplifier.

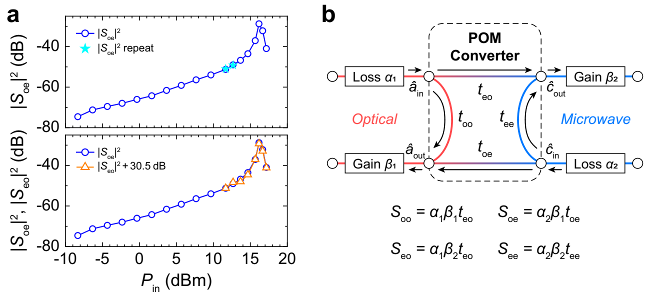

Supplementary Figure 3. **Measurement repeatability and efficiency calibration principle.** **a** Directly measured bidirectional conversion signals at different pump power $P_{\rm in}$. The upper panel shows repeated measurement of $S_{\rm oe}$ at lower powers after sweeping to the highest applied pump power. The results overlap very well with the original data without hysteresis effect. The lower panel shows the consistent power dependence of $S_{\rm oe}$ and $S_{\rm eo}$. The $|S_{\rm eo}|^2$ curve is manually shifted up by 30.5 dB for comparison purpose. **b** Signal flowing diagram showing the relation between the directly measured scattering matrix elements S_{ij} and the intrinsic on-chip elements t_{ij}, $(i, j = \text{o,e})$. The gain and loss factors (α's and β's) can be calibrated out together to obtain the intrinsic conversion efficiency.

Supplementary Note 4. Measurement repeatability and efficiency calibration

The bidirectional conversion signals in our experiment are highly repeatable within the largest applied pump power at 17.2 dBm. As indicated as cyan stars in upper panel of Fig. 3a, we repeat the $S_{\rm oe}$ measurement at low powers after sweep up to highest pump power. It can be seen that even after a few hours of delay between measurements, the results still overlap very well with previous data, confirming no hysteresis effects. This indicates that during our experiment, the "Ouroboros" remains well in its superconducting state with stable and repeatable resonant frequency even in presence of the pump induced heating and the external magnetic bias field. In addition, we plot the measured $S_{\rm eo}$ at different pump powers in the lower panel of Fig. 3a to show consistent power dependence as the $S_{\rm oe}$ ($|S_{\rm eo}|^2$ is manually shifted by 30.5 dB for comparison purpose), revealing the bidirectional and reciprocal nature of the M-O photon conversion.

The conversion efficiency is calibrated by measuring the full spectra of the scattering matrix elements, following the procedure described in [1]. The principle of the calibration is illustrated in Fig. 3b. The directly measured scattering matrix elements S_{ij} are proportional to the intrinsic elements of the converter up to a constant gain or loss factor. Although it is difficult to calibrate individual gain or loss factor along each path, they can be canceled out together to obtain the intrinsic conversion efficiency since conversion is reciprocal. The on-chip efficiency (peak of the spectrum at $\omega = \omega_{\rm m}$) can be obtained by

$$\eta_0 = |t_{\rm oe}[\omega_{\rm m}]t_{\rm eo}[\omega_{\rm m}]| = \frac{|S_{\rm oe}[\omega_{\rm m}]S_{\rm eo}[\omega_{\rm m}]|}{|S_{\rm oo,bg}[\omega_{\rm m}]S_{\rm ee,bg}[\omega_{\rm m}]|}, \tag{S2}$$

where $S_{\rm oo,bg}$ and $S_{\rm ee,bg}$ denote the background of the reflection spectra $S_{\rm oo}$ and $S_{\rm ee}$ without resonance, namely, $\alpha_1\beta_1$ and $\alpha_2\beta_2$. By fitting the reflection and conversion spectra at the optimal pump power of 16.2 dBm, we extract $|S_{\rm oo,bg}[\omega_{\rm m}]|^2 = (4.27\pm0.02)\times10^{-2}$, $|S_{\rm ee,bg}[\omega_{\rm m}]|^2 = (6.44\pm0.24)\times10^{-2}$, $|S_{\rm oe}[\omega_{\rm m}]|^2 = (1.29\pm0.04)\times10^{-3}$, $|S_{\rm eo}[\omega_{\rm m}]|^2 = (1.15\pm0.02)\times10^{-6}$. Using Eq. (S2) and the propagation of uncertainty, we can calibrate the highest on-chip conversion efficiency to be $(7.3 \pm 0.2) \times 10^{-4}$, corresponding to an internal efficiency of $(5.8 \pm 0.2)\%$.

In our experiment, the microwave and the optical input powers are set to be low enough to avoid any nonlinear effects. As an example, we give the typical signal power levels that are used when the optical pump power is 16 dBm with the highest conversion efficiency. For microwave-to-optical conversion, the typical microwave input power is around -70 dBm (photon number rate $\sim10^{13}$ Hz), and the converted optical power is around -55 dBm (photon number rate $\sim10^{10}$ Hz). For optical-to-microwave conversion, since an optical single-sideband modulator was used to generate the optical input, the sideband power is typically controlled to be ~50 dB lower than the pump. So at 16 dBm pump power, the optical input power is around -35 dBm (photon number rate $\sim10^{12}$ Hz), and the converted microwave power is around -110 dBm (photon number rate $\sim10^9$ Hz).

Supplementary Note 5. Pulse response and conversion spectrum

A. Optical cavity photon response

We first calculate the response of the classical intra-cavity field $\alpha(t)$ under a pulse pump input $\alpha_{\rm in}(t)$, which is related to the input pump power via $P_{\rm in}(t)=\hbar\omega_{\rm o}|\alpha_{\rm in}(t)|^2$. To first order approximation, the optomechanical backaction can be neglected and α simply follows

$$\dot{\alpha}(t)=\Big(i\Delta_{\rm o}-\frac{\kappa_{\rm o}}{2}\Big)\alpha(t)+\sqrt{\kappa_{\rm o,c}}\alpha_{\rm in}(t). \tag{S3}$$

If the rising edge of the input is a perfect step function, namely, $\alpha_{\rm in}(t)=0$ when $t<0$ and $\alpha_{\rm in}(t)=\alpha_0$ is constant when $t\geq 0$, the intra-cavity photon number can be solved as ($t\geq 0$)

$$n_{\rm cav}(t)\equiv|\alpha(t)|^2=\frac{\kappa_{\rm o,c}\left|\alpha_0^2\right|}{\Delta_{\rm o}^2+\kappa_{\rm o}^2/4}\left[1+e^{-\kappa_{\rm o}t}-2\cos{(\Delta_{\rm o}t)}e^{-\frac{\kappa_{\rm o}}{2}t}\right], \tag{S4}$$

which reaches steady state with a time constant $1/\kappa_{\rm o}$. Similarly, the falling edge will have the same response time. The cosine oscillation term in solution Eq. (S4) is due to the perfectly sharp step edge of the input which doesn't exist in experiment. Therefore, in numerical calculation in Fig. 3b of the main text, we used a Gauss error function for a smoother input, instead of a step function, to reduce the artificial oscillation.

B. Conversion signal response

Now we analyze the pulse response of the conversion photon. Due to the fast response of the $n_{\rm cav}(t)$, the optomechanical coupling $g_{\rm om}(t)\equiv g_{\rm om,0}\sqrt{n_{\rm cav}(t)}$ can be simply treated as a step function. In other words, the pump pulse serves as a fast switch to quickly turn on and off the optomechanical coupling. In the resolved-sideband limit ($\omega_{\rm m}\gg\kappa_{\rm o}$), the Heisenberg equations of motion of the intra-cavity field $\mathbf{a}(t)=(\hat{a},\hat{c},\hat{b})^T$ can be written as [2]

$$\dot{\mathbf{a}}(t)=\mathbf{A}\mathbf{a}(t)+\mathbf{B}\mathbf{a_{in}}(t), \tag{S5}$$

where $\mathbf{a_{in}}(t)=(a_{\rm in1},a_{\rm in2},c_{\rm in1},c_{\rm in2},b_{\rm in2})^T$ is the input term. The subscript (1, 2) denotes the coupling or the intrinsic (noise) port, respectively. Matrices $\mathbf{A}$ and $\mathbf{B}$ are given by

$$\mathbf{A}=\begin{pmatrix} i\Delta_{\rm o}-\frac{\kappa_{\rm o}}{2} & 0 & ig_{\rm om}(t)\\ 0 & -i\omega_{\rm e}-\frac{\kappa_{\rm e}}{2} & ig_{\rm em}\\ ig_{\rm om}(t) & ig_{\rm em} & -i\omega_{\rm m}-\frac{\kappa_{\rm m}}{2}\end{pmatrix},\quad \mathbf{B}=\begin{pmatrix}\sqrt{\kappa_{\rm o,c}} & \sqrt{\kappa_{\rm o,i}} & 0 & 0 & 0\\ 0 & 0 & \sqrt{\kappa_{\rm e,c}} & \sqrt{\kappa_{\rm e,i}} & 0\\ 0 & 0 & 0 & 0 & \sqrt{\kappa_{\rm m}}\end{pmatrix}. \tag{S6}$$

Combining with the input-output relation

$$\mathbf{a_{out}}(t)=\mathbf{B}^T\mathbf{a}(t)-\mathbf{a_{in}}(t) \tag{S7}$$

and setting the noise input terms to be zero, we can numerically calculate the temporal profiles of the conversion signals as plotted in Fig. 3b of the main text.

The physical understanding of the transient conversion response is as follows. In general, characteristic response time of a cavity is determined by its total energy exchange rate (including all dissipation and coupling channels). For the mechanical mode, besides its own intrinsic dissipation $\kappa_{\rm m}$, the electromechanical coupling provides an effective energy transfer rate $\sim\frac{4g_{\rm em}^2}{\kappa_{\rm o}}=C_{\rm em}\kappa_{\rm m}$. When $g_{\rm om}$ is turned on by the pump pulse, the effective optomechanical energy transfer rate is $\sim C_{\rm om}\kappa_{\rm m}$. Therefore, the mechanical total energy exchange rate is $\Gamma_{\rm m}\sim(1+C_{\rm em}+C_{\rm om})\kappa_{\rm m}$, which results in a response time $\sim 1/\Gamma_{\rm m}\ll 1\,\mu$s. Similarly, the microwave total rate is $\Gamma_{\rm e}\sim(1+C_{\rm em})\kappa_{\rm e}$ and $1/\Gamma_{\rm e}\ll 1\,\mu$s. Therefore, as confirmed by the numerical results, the conversion signals can quickly reach steady state during the 3-μs pump pulse. It is worth pointing out that, due to the pump induced heating, the temperature of system will gradually increase during the pulse. However, since the thermal dynamics is a much slower process compared with the photon conversion, it is a good approximation to treat the temperature and resonance frequencies as constants when analyzing the conversion dynamics. In our experiment, the measurement time constant of each temporal data point is set to be as short as 30 ns. During such short period of time, the temperature change of the system can be neglected, and hence, the conversion process can be treated as a quasi-steady state.

C. Conversion spectrum

Solving Eq. (S5) and (S7) in the frequency domain, we can define the scattering matrix $\mathbf{S}[\omega]$ from $\mathbf{a_{out}}[\omega] = \mathbf{S}[\omega]\mathbf{a_{in}}[\omega]$, which can be expressed as

$$\mathbf{S}[\omega] = \mathbf{B}^T[-i\omega\mathbf{I}_3 - \mathbf{A}]^{-1}\mathbf{B} - \mathbf{I}_5, \tag{S8}$$

where $\mathbf{I}_3$ and $\mathbf{I}_5$ are 3×3 and 5×5 identity matrix, respectively. The (1,3) and the (3,1) elements of $\mathbf{S}$ correspond to the microwave-to-optical and the optical-to-microwave conversion coefficients ($t_{\rm oe}$ and $t_{\rm eo}$ in the main text), respectively; namely, $\mathrm{S}_{1,3}[\omega] \equiv a_{\rm out1}[\omega]/c_{\rm in1}[\omega] = t_{\rm oe}[\omega]$ and $\mathrm{S}_{3,1}[\omega] \equiv c_{\rm out1}[\omega]/a_{\rm in1}[\omega] = t_{\rm eo}[\omega]$. The M-O photon conversion spectrum $\eta[\omega] \equiv |t_{\rm oe}[\omega]|^2 = |t_{\rm eo}[\omega]|^2$ can be obtained as

$$\eta[\omega] = \frac{\kappa_{\rm e,c}}{\kappa_{\rm e}}\frac{\kappa_{\rm o,c}}{\kappa_{\rm o}}\frac{4C_{\rm em}C_{\rm om}}{\left|C_{\rm em}\left(1-i\frac{\omega+\Delta_{\rm o}}{\kappa_{\rm o}/2}\right)+\left(1-i\frac{\omega-\omega_{\rm e}}{\kappa_{\rm e}/2}\right)\left[C_{\rm om}+\left(1-i\frac{\omega+\Delta_{\rm o}}{\kappa_{\rm o}/2}\right)\left(1-i\frac{\omega-\omega_{\rm m}}{\kappa_{\rm m}/2}\right)\right]\right|^2}, \tag{S9}$$

which reduces to Eq. (2) in the main text when $\Delta_{\rm o} = -\omega_{\rm m}$ and $\omega = \omega_{\rm m}$.

Supplementary Note 6. Noise analysis

The added noise during the photon conversion process can be analyzed by calculating the power spectrum density (PSD) of the output fields. For a field $\hat{x}(t)$, the power spectrum density is given by $S_{\rm x}[\omega] = \frac{1}{2\pi}\int_{-\infty}^{+\infty} {\rm d}\omega' \langle \hat{x}^\dagger[-\omega]\hat{x}[\omega']\rangle$, where $\hat{x}[\omega]$ is the Fourier transform of $\hat{x}(t)$. The input thermal noise terms satisfy relations

$$\begin{aligned}
\langle \hat{b}_{\rm in2}[\omega]\hat{b}^\dagger_{\rm in2}[\omega']\rangle &= (\bar{n}_{\rm th,m}+1)2\pi\delta(\omega+\omega'),\\
\langle \hat{b}^\dagger_{\rm in2}[\omega]\hat{b}_{\rm in2}[\omega']\rangle &= \bar{n}_{\rm th,m}2\pi\delta(\omega+\omega'),\\
\langle \hat{c}_{\rm in2}[\omega]\hat{c}^\dagger_{\rm in2}[\omega']\rangle &= (\bar{n}_{\rm th,e}+1)2\pi\delta(\omega+\omega'),\\
\langle \hat{c}^\dagger_{\rm in2}[\omega]\hat{c}_{\rm in2}[\omega']\rangle &= \bar{n}_{\rm th,e}2\pi\delta(\omega+\omega'),
\end{aligned} \tag{S10}$$

with

$$\bar{n}_{\rm th,m} = \frac{1}{e^{\hbar\omega_{\rm m}/k_{\rm B}T_{\rm m}}-1}, \quad \bar{n}_{\rm th,e} = \frac{1}{e^{\hbar\omega_{\rm e}/k_{\rm B}T_{\rm e}}-1}. \tag{S11}$$

Here, $T_{\rm m}$ and $T_{\rm e}$ are the temperatures of the intrinsic mechanical and microwave thermal baths, respectively. Note that we have neglected the thermal noise of the optical mode since it will be at the quantum ground state even at room temperature.

For **microwave-to-optical conversion (upconversion)**, the input fields are the microwave signal $\hat{c}_{\rm in1}$, the microwave noise $\hat{c}_{\rm in2}$, and the mechanical noise $\hat{b}_{\rm in2}$. Then the optical output PSD can be expressed as

$$\begin{aligned}
S_{\rm aout1}[\omega] = &|\mathrm{S}_{1,3}|^2\frac{1}{2\pi}\int_{-\infty}^{+\infty}{\rm d}\omega'\langle\hat{c}^\dagger_{\rm in1}[-\omega]\hat{c}_{\rm in1}[\omega']\rangle + |\mathrm{S}_{1,4}|^2\frac{1}{2\pi}\int_{-\infty}^{+\infty}{\rm d}\omega'\langle\hat{c}^\dagger_{\rm in2}[-\omega]\hat{c}_{\rm in2}[\omega']\rangle\\
&+|\mathrm{S}_{1,5}|^2\frac{1}{2\pi}\int_{-\infty}^{+\infty}{\rm d}\omega'\langle\hat{b}^\dagger_{\rm in2}[-\omega]\hat{b}_{\rm in2}[\omega']\rangle.
\end{aligned} \tag{S12}$$

The first term is the converted optical signal, the second and the third terms are noise. The *added noise* of the converter is defined as the noise referred to the input. Plugging in Eq. (S10), we can get the microwave added noise $n_{\rm up,e}$ and the mechanical added noise $n_{\rm up,m}$ as

$$n_{\rm up,e} = \frac{|\mathrm{S}_{1,4}|^2}{|\mathrm{S}_{1,3}|^2}\bar{n}_{\rm th,e} = \frac{\kappa_{\rm e,i}}{\kappa_{\rm e,c}}\bar{n}_{\rm th,e}, \tag{S13}$$

$$n_{\rm up,m} = \frac{|\mathrm{S}_{1,5}|^2}{|\mathrm{S}_{1,3}|^2}\bar{n}_{\rm th,m} = \frac{\left[\left(\frac{\kappa_{\rm e}}{2}\right)^2+(\omega-\omega_{\rm e})^2\right]\kappa_{\rm m}}{g_{\rm em}^2\kappa_{\rm e,c}}\bar{n}_{\rm th,m}. \tag{S14}$$

In our experiment, the microwave thermal bath is estimated to have only a few photons ($\bar{n}_{\mathrm{th,e}} \approx 1.6$ at 1 K, and 5.6 at 3 K). Therefore $n_{\mathrm{up,e}}$ can be easily suppressed to below one when the microwave coupling port is very over-coupled ($\kappa_{\mathrm{e,i}} \ll \kappa_{\mathrm{e,c}}$). For the mechanical added noise, when $\omega = \omega_{\mathrm{e}} = \omega_{\mathrm{m}}$, we have $n_{\mathrm{up,m}} = \frac{1}{C_{\mathrm{em}}}\frac{\kappa_{\mathrm{e}}}{\kappa_{\mathrm{e,c}}}\bar{n}_{\mathrm{th,m}}$. Namely, a large C_{em} and the over-coupling condition can suppress the mechanical added noise even if device is physically in a "hot" thermal bath. These theoretical analyses, in fact, reveal the effect of radiative cooling—that is by over coupling the microwave mode to a cold bath, the electromechanical modes can be cooled to quantum ground state despite the large thermal occupancy of their physical environment.

For **optical-to-microwave conversion (downconversion)**, the input fields are the optical signal $\hat{a}_{\mathrm{in1}}$, the mechanical noise $\hat{b}_{\mathrm{in2}}$, and the microwave noises from both the intrinsic bath $\hat{c}_{\mathrm{in2}}$ and the coupling port $\hat{c}_{\mathrm{in1}}$. Hence, the microwave output PSD is

$$\begin{aligned} S_{\mathrm{cout1}}[\omega] = {} & |\mathrm{S}_{3,1}|^2 \frac{1}{2\pi}\int_{-\infty}^{+\infty} \mathrm{d}\omega' \langle \hat{a}^{\dagger}_{\mathrm{in1}}[-\omega]\hat{a}_{\mathrm{in1}}[\omega']\rangle + |\mathrm{S}_{3,5}|^2 \frac{1}{2\pi}\int_{-\infty}^{+\infty} \mathrm{d}\omega' \langle \hat{b}^{\dagger}_{\mathrm{in2}}[-\omega]\hat{b}_{\mathrm{in2}}[\omega']\rangle \\ & + |\mathrm{S}_{3,4}|^2 \frac{1}{2\pi}\int_{-\infty}^{+\infty} \mathrm{d}\omega' \langle \hat{c}^{\dagger}_{\mathrm{in2}}[-\omega]\hat{c}_{\mathrm{in2}}[\omega']\rangle + |\mathrm{S}_{3,3}|^2 \frac{1}{2\pi}\int_{-\infty}^{+\infty} \mathrm{d}\omega' \langle \hat{c}^{\dagger}_{\mathrm{in1}}[-\omega]\hat{c}_{\mathrm{in1}}[\omega']\rangle. \end{aligned} \tag{S15}$$

The first term is the converted microwave signal, the second and the third terms are the mechanical and the microwave noises. The last term is the noise coming in from the microwave input port, which then gets reflected and comes out as part of the microwave output. For practical quantum operation, a circulator is needed to separate the input and the output fields of the microwave port. The microwave input should be thermalized at ∼mK environment to make sure this last term in Eq. (S15) contributes zero noise. This is also consistent with our idea and experimental implementation of the radiative cooling [3]. Similarly, the microwave and the mechanical added noise during the downconversion can be obtained as

$$n_{\mathrm{down,e}} = \frac{|\mathrm{S}_{3,4}|^2}{|\mathrm{S}_{3,1}|^2}\bar{n}_{\mathrm{th,e}} = \frac{\left|g_{\mathrm{om}}^2 + \left[\frac{\kappa_{\mathrm{o}}}{2} - i(\omega+\Delta_{\mathrm{o}})\right]\left[\frac{\kappa_{\mathrm{m}}}{2} - i(\omega-\omega_{\mathrm{m}})\right]\right|^2 \kappa_{\mathrm{e,i}}}{g_{\mathrm{em}}^2 g_{\mathrm{om}}^2 \kappa_{\mathrm{o,c}}}\bar{n}_{\mathrm{th,e}}, \tag{S16}$$

$$n_{\mathrm{down,m}} = \frac{|\mathrm{S}_{3,5}|^2}{|\mathrm{S}_{3,1}|^2}\bar{n}_{\mathrm{th,m}} = \frac{\left[\left(\frac{\kappa_{\mathrm{o}}}{2}\right)^2 + (\omega+\Delta_{\mathrm{o}})^2\right]\kappa_{\mathrm{m}}}{g_{\mathrm{om}}^2 \kappa_{\mathrm{o,c}}}\bar{n}_{\mathrm{th,m}}. \tag{S17}$$

We see that the thermal noises contribute differently in different conversion directions. When $\omega = \omega_{\mathrm{e}} = \omega_{\mathrm{m}} = -\Delta_{\mathrm{o}}$, the microwave added noise simplifies to $n_{\mathrm{down,e}} = \frac{(C_{\mathrm{om}}+1)^2}{C_{\mathrm{om}}C_{\mathrm{em}}}\frac{\kappa_{\mathrm{o}}}{\kappa_{\mathrm{o,c}}}\frac{\kappa_{\mathrm{e,i}}}{\kappa_{\mathrm{e}}}\bar{n}_{\mathrm{th,e}}$. As we have discussed in the main text, a high conversion efficiency requires large and matched C_{om} and C_{em}, so the first factor would be around one. An over-coupled optical port will reduce the second factor to close to one. Therefore, it is again very crucial to over couple the microwave port ($\kappa_{\mathrm{e,i}}/\kappa_{\mathrm{e}} \ll 1$) to suppress $\bar{n}_{\mathrm{th,e}}$. The mechanical added noise at $\omega = \omega_{\mathrm{m}} = -\Delta_{\mathrm{o}}$ becomes $n_{\mathrm{down,m}} = \frac{1}{C_{\mathrm{om}}}\frac{\kappa_{\mathrm{o}}}{\kappa_{\mathrm{o,c}}}\bar{n}_{\mathrm{th,m}}$. Hence, it is important to have a large C_{om} to suppress the mechanical noise during the optical-to-microwave photon conversion.

[1] Fan, L., Zou, C.-L., Cheng, R., Guo, X., Han, X., Gong, Z., Wang, S. & Tang, H. X. Superconducting cavity electro-optics: a platform for coherent photon conversion between superconducting and photonic circuits. *Sci. Adv.* **4**, eaar4994 (2018).

[2] Zhong, C., Wang, Z., Zou, C.-L., Zhang, M., Han, X., Fu, W., Xu, M., Shankar, S., Devoret, M. H., Tang, H. X. & Jiang, L. Proposal for heralded generation and detection of entangled microwave–optical photon pairs. *Phys. Rev. Lett.* **124**, 010511 (2020).

[3] Xu, M., Han, X., Zou, C.-L., Fu, W., Xu, Y., Zhong, C., Jiang, L. & Tang, H. X. Radiative cooling of a superconducting resonator. *Phys. Rev. Lett.* **124**, 033602 (2020).
